# Constipation and Its Relation to Pelvic Disease in Young Women

**Published:** 1891-05

**Authors:** E. H. Root

**Affiliations:** Chicago., Ill.; Professor of Hygiene and Medical Jurisprudence in the Woman’s Medical College


### CONSTIPATION AND ITS RELATION TO PELVIC DISEASE IN YOUNG WOMEN.

BY E. H, ROOT. M. D., CHICAGO., ILL.

Professor of Hygiene and Medical Jurisprudence in the Woman's Medical College.

Constipation, long continued, gives rise to a train of symptoms that present themselves for relief in the daily practice of every active physician. Nor does he find any one cause of ill-health so difficult to remove. The habit is generally well established before he sees the patient, and she comes for the relief of resulting conditions and symptoms.

The habit of continued and obstinate constipation is largely the result of a careless disregard of physiological functions, and is entirely lost sight of by the patient, as a possible cause of ill health and physical discomfort. It begins, too frequently, in childhood, continuing into womanhood and accompanying its victims to the grave. Many of the symptoms point to pelvic disease ; pain in the back, pain in the top and back of the head, a feeling of weight in the limbs, dysmenorrhoea and leucorrhoea, subjecting the young patient to local treatment, when changed modes of dress and life with the relief of constipation, would restore health.

Still, it is very evident that constipation does act as a most active factor in the production of pelvic disease in women ; for, where endocervicitis and even retroversion exist, rapid improvement is made as soon as constipation is relieved.

The causes of constipation are varied and arise frequently in childhood from faulty diet, dress and neglect of elimination. Again, it occurs at the time of puberty, when the transition from girl to womanhood gives rise to faulty nutrition and consequent relaxation of all the bodily tissues. Tight clothing, with high pressure modes of life, together with the neglect of daily retirement, will at this time of the young girl's life, establish the habit of constipation fatal to her future health and comfort.

Anaemia, red, spongy gums, greasy and coarse skin, acne, offensive breath, chronic pharyngitis, naso-pharyngeal catarrhs and rheumatic pains co-exist singly or altogether with, and as a result of constipation, and where they do so co-exist, pelvic disorders are rarely absent in women who have reached the ages of between 21 and 30 years.

Pelvic disease results from constipation through pressure. The filled and distended sigmoid flexure and rectum press upon blood vessels, nerves and muscles, exerting direct and reflex influences upon the functional activities of the whole economy. The sigmoid flexure lies frequently against the posterior surface of the broad ligament (Charpentier.) The left ovary and broad ligament, with their nerves and blood vessels, come more directly under the influence of mechanical disturbances, hence left ovarian tenderness and pain are more frequent than right. Pressure produces dysmenorrhoea by increasing the normal congestion of menstruation to a pathological congestion, and consequent hyperaesthesia of the pelvic nerves, and by displacements. Such abnormal congestion is never wholly cleared away by the normal ebb and flow of the pelvic circulation, and disease of the endometrium, in part (endocervicitis) or complete (endometritis), is the result.

Constipation can aid the establishment of ovarian and uterine disease and displacements indirectly, through the malnutrition and consequent weakening of the tissues incident to poor elimination of waste products. Once the uterine supports are weakened, the proper relations of the pelvic viscera are destroyed, and the weight and pressure of the intestinal loops, distended or empty, will serve to fix the displaced uterus and ovaries in their abnormal position. The uterus, having a marked faculty of reaction upon the cerebro-spinal system, a train of distressing symptoms arise, and when associated with artificial modes of life and surroundings, together with inherited strumous or neurotic tendencies, a crippled motherhood and physical ruin is certain.

The sigmoid flexure, with loops of small intestines, may give rise to confusion and even mistakes in the diagnosis of pelvic disease.

If we consider the development and adult anatomy of the intestine, we cannot dismiss the idea that anomalies and displacements of its various sections may occur. It is needless to speak here of the displacement downward of the transverse colon; still, we may state that when so displaced and loaded with faeces it may lead to a mistake in diagnosis.

But what I believe to be of more frequent occurrence is confusion arising from the sigmoid flexure. In making a vaginal examination it can be readily felt if behind the uterus, and when empty is more difficult to recognize than when filled.

Let us consider, for a moment, the anatomical relations and development of this section of the intestinal tract. "The sigmoid flexure of the colon, situated in the left iliac fossa, consists of a double bending of the intestine upon itself in the form of the letter "S," or, as some anatomists state, the form of the Greek omega lying upon its side "ꞷ" immediately before it becomes continuous with the rectum at the margin of the pelvis opposite to the left sacro-iliac articulation. It is attached by a distinct meso-colon to the iliac fossa, and is very movable, falling into the pelvis when the bladder is empty. It is placed immediately behind the anterior wall of the abdomen, and is separated from the uterus by some convolutions of the small intestine. In normal conditions and relations the sigmoid flexure may be above the uterus. But normal conditions have no need for medical investigation, and only the abnormal present themselves.

Of the development of the sigmoid flexure we learn that "the large intestine of the foetus at birth, is comparatively longer than that of the adult. In the infant, it is always nearly three times the length of the entire body; in the adult it is but twice the length of the body;" and "as the colon ascendens is very short, and the transversum is not long, the principal part of the excessive length falls upon the sigmoid flexure" with a length varying from 8 to 30 ctms. (Jacobi). This length gives rise to several convolutions instead of the simple sigmoid flexure. Its position also varies from left to right. One author says: "In one case in six, it is on the right side." Another states that in four per centum of the cases examined, it was more in the median line and more to the left than the right side, and Jacobi states that his own experience has taught him to look on the right side of infants for the flexure.

You ask, What has this to do with adult conditions? It may have much to do with them. With the wide variances in length and position in infancy it seems more than reasonable to assume that corresponding variances exist in adults.

Post mortems reveal a sufficient number of such variations to make it more than probable that they exist to a considerable extent. Constipation will aid this flexure, if in a perfectly normal condition as to length and position, to seek the pelvic cavity. Constant constipation will not only dilate the viscus, but it will drag it down, stretching its meso-colon.

The laxness of fiber, so often found in women suffering from pelvic inflammations and constipation, is an additional factor in the prolapsing. It may fall behind the uterus, and when a variation as to length and position is present, it will be felt at the median line or in the right side. The examining finger coming in contact with it in these positions, is ready to assume the presence of serious disease products. It may fall in front of the uterus, with other loops of small intestines, and hold the retroverted uterus securely in the hollow of the sacrum by means of its superincumbent weight.

Adhesions are frequently diagnosticated, and laparotomy, when made, proves their non-existence. The exploring finger of the operator having in all probability disturbed the relations of the dependent loops, and they are lost sight of as the cause of the mistake. When there is any resistance felt to the upward and forward movement of the retroverted uterus, the probable presence of superincumbent loops of intestine should not be lost sight of, and the operation should be done only in the knee—chest or Sim's position. To do otherwise is to render the operation painful and hazardous, and always unsuccessful, for the uterus, on removal of the force, falls immediately back in its old position.

CASE 1. A young woman, single, aged 23, came to me for the relief of dysmenorrhoea. Constipation had existed for years; fibers lax; anaemic and greatly debilitated. On examination I found tenderness of the uterus and tenderness throughout the pelvis. The uterus was displaced slightly backward and quite strongly prolapsed. Behind the uterus, extending well to the right side, I found a large, irregular, nodulated hard mass. Pressure upon it caused pain, and feeling it through the intervening tissues it gave to the examining finger a rather startling impression; but following the mass toward the left, I was led to pass the finger over the rectum, in which I found two or three nodes, hard and free from surrounding infiltrations. I concluded I had a case of elongated and dilated sigmoid flexure filled with hardened excreta.

I ceased further manipulation and directed the patient to take a compound cathartic pill, and to aid its action, the following morning with enema, and then to come again. On a second examination I found the post-uterine tumor (?) gone, tenderness abated and the uterus in a nearly normal position. The menstrual pain was variable in severity and duration, and I inferred that the pain arose from pressure and pelvic congestion induced by obstruction of the pelvic circulation.

Case II. A young woman, aged 21, single, blonde, of pale, waxy complexion; menstruation painful and often accompanied by hysterical attacks. She suffered from indigestion, and a persistent cough had led her friends to fear phthisis. The treatment she received, though of the best, did not stop the cough, and she remained pale and weak; and as the condition of the lungs did not warrant the idea of established phthisis, she was sent to me for a pelvic examination.

I found a retroverted uterus, endocervicitis, erosion of the cervix, and a prolapsed ovary. The displacement was not reducible, and seemed fixed in the hollow of the sacrum. Placing the patient in Sim's position, then to the knee-chest position, I found the uterus free.

At a subsequent examination I found an enlargement behind and above the uterus that I had not found at any previous visit. Its presence gave me some uneasiness, as constipation was not complained of this time, nor did the enlargement have the appearance of being a distended loop of the bowel. While trying to move the uterus forward, it was set free with a gurgling sound and sensation, felt by the patient as well as by myself. The uterus assumed the normal position, the enlargement had disappeared and I felt satisfied of its character.

Menstrual pain was also variable in this case. I advised the patient to take an enema or a Seidlitz powder just before the onset of the flow, and to resort to the knee-chest position several times a day during the first of the flow, and was rewarded by marked relief in suffering. Under general and local treatment the anaema disappeared with the endocervicitis. There was no more cough, and the stomach consented to perform its functions.

CASE III, demonstrates the confusion an anomalous sigmoid curve can give rise to. This patient was brought to the Hospital for Women and Children fcr care by Dr. Addison H. Foster. Dr. William E. Clarke saw the case with him. No one knowing these gentlemen will, for a moment, question their ability to make a diagnosis.

Pelvic suppurative inflammation, with abdominal peritonitis, was certain.

A rounded tumor was felt in the vagina to the right side; while just above Poupart's ligament, external manipulation revealed, on the right side, a tumor. It could not be determined if that felt in the vagina was continuous with and a part of the one felt externally. The bowels moved every day, the evacuations being normal in amount and consistency. The patient died, and a post mortem cleared up all doubt as to the character of the tumor felt by external palpation. It was the superior curve of the sigmoid flexure. The intestine extended from left to right almost tranversely, filled to distension throughout its length. The right extremity lay behind the anterior wal of the abdomen; hence the tumor. It could not be followed by external manipulation, as its middle portion was covered with loops of small intestine and omentum, firmly bound by adhesions. The lower curve sank more into the pelvis and was lost in a mass of adhesions and pus cavities.

The patient had been constipated during the greater part of life, but at the time of entering the hospital was apparently free from it, which led still more to the confusion.

This matter of constipation does not receive the attention it should. Its prevention in children should be the concern of mother and teacher, and that from an intelligent comprehension of the physiological functions of the body; and where neglect and ignorance exist, the patient's attention should be arrested by the counsel and advice of the physician.

